# Quercetin extracted from Sophora japonica flower improves growth performance, nutrient digestibility, cecal microbiota, organ indexes, and breast quality in broiler chicks

**DOI:** 10.5713/ab.21.0331

**Published:** 2022-01-04

**Authors:** De Xin Dang, Sungbo Cho, Huan Wang, Woo Jeong Seok, Jung Heun Ha, In Ho Kim

**Affiliations:** 1 Department of Animal Resource and Science, Dankook University, Cheonan 31116, Korea; 2 School of Mongolian Medicine, Inner Mongolia University for Nationalities, Tongliao, Inner Mongolia Autonomous Region 028000, China; 3 School of Biology and Food Engineering, Chuzhou University, Chuzhou, 239000, China; 4 Department of Food Science and Nutrition, Dankook University, Cheonan 31116, Korea; 5 Research Center for Industrialization of Natural Neutralization, Dankook University, Cheonan 31116, Korea

**Keywords:** Broiler Chick, Meat Quality, Nutrient Digestibility, Quercetin, *Sophora japonica*

## Abstract

**Objective:**

The objective of this study was to evaluate the effects of supplementing quercetin extracted from *Sophora japonica* flower (QS) to the diet of broiler chicks on their growth performance, apparent nutrient digestibility, cecal microbiota, serum lipid profiles, relative organ weight, and breast muscle quality.

**Methods:**

A total of 1,088 1-day-old broiler chicks (mixed sex) were randomly assigned to four groups based on the initial body weight (43.00±0.29 g). The experimental period was 35 days (starter, days 0 to 7; grower, days 7 to 21; finisher, days 21 to 35). There were 17 replicate cages per treatment and 16 birds per cage. Dietary treatments consisted of birds receiving basal diet without quercetin as the control group and treatment groups consisted of birds fed basal diet supplemented with 0.2, 0.4, or 0.6 g/kg QS.

**Results:**

With the increase of the QS dosage, body weight gain during days 0 to 7 (p = 0.021), 7 to 21 (p = 0.010), and 1 to 35 (p = 0.045), feed intake during days 0 to 7 (p = 0.037) and 1 to 35 (p = 0.025), apparent dry matter digestibility (p = 0.008), apparent energy retention (p = 0.004), cecal lactic acid bacteria counts (p = 0.023), the relative weight of breast muscle (p = 0.014), pH value from breast muscle (p<0.001), and the water holding capacity of breast muscle (p = 0.012) increased linearly, whereas the drip loss from breast muscle (p = 0.001) decreased linearly.

**Conclusion:**

The addition of QS in the diet of broiler chicks had positive effects on the breast muscle yield and breast muscle quality, and improved the dry matter digestibility and energy retention by increasing cecal beneficial bacteria counts, thus improving growth performance.

## INTRODUCTION

Flavonoids have a variety of biological activities and are widely used in the modern livestock industry [1–3]. It has been reported that flavonoid supplementation had beneficial effects on the improvement of growth performance, nutrient digestibility, intestinal microbiota community, immune status, and antioxidant status in poultry [4,5].

Quercetin is a kind of flavonoid compound which is widely found in fruits or vegetables [6]. Quercetin has been widely reported for its excellent biological properties such as antibacterial, antioxidant, gut health improver, growth promoter, and immunomodulatory [7–10]. The sources of quercetin include chemical synthesis or plant extraction. It has been reported that dietary supplementation of chemically synthesized quercetin was not beneficial to the growth performance in broiler chicks [8,11–16]. Therefore, modern poultry husbandry focused on the quercetin derived from plants.

Zhang and Kim [17] reported that dietary supplementation of 0.25, 0.50, or 1.00 g/kg plant-derived quercetin improved growth performance, cecal microbiota community, and relative organ weight in broiler chicks. Abid et al [18] reported that feeding broiler chicks with 0.1, 0.2, or 0.3 g/kg quercetin extracted from locust trees containing diet improved the feed efficiency. Sohaib et al [19] reported that broiler chicks fed the diet supplemented with 0.1, 0.2, or 0.3 g/kg plant-derived quercetin improved growth performance. However, no studies have evaluated the effects of dietary supplementation of quercetin extracted from the flower of *Sophora japonica* (QS) on growth performance in broiler chicks.

We hypothesized that supplementing QS to the diet of broiler chicks could improve the apparent nutrient digestibility by regulating the cecal microbiota community, thus improving growth performance. The objective of this study was to evaluate the effects of QS supplementation on growth performance, apparent nutrient digestibility, cecal microbiota, serum lipid profiles, relative organ weight, and breast muscle quality in broiler chicks.

## MATERIALS AND METHODS

The experiment was conducted under the supervision of the Animal Care and Use Committee of Dankook University (Cheonan, South Korea), and the relevant experimental protocol has been approved (No. DK-1-2032).

### Animals, diets, and experimental design

A total of 1,088 day-old (as hatched) Ross 308 broiler chicks (mixed sex) were randomly assigned to four groups based on the initial body weight (43.00±0.29 g). There were 17 replicate cages per treatment and 16 birds per cage. The experimental period was 35 days, including starter period (days 0 to 7), grower period (days 7 to 21), and finisher period (days 21 to 35). The dietary treatments used included: i) basal diet (no additive, control group), ii) basal diet including 0.2 g/kg of QS, iii) basal diet including 0.4 g/kg QS, and iv) basal diet including 0.6 g/kg QS. The commercial quercetin additive (Synergen, 190, Sinheung, Bucheon, Gyeonggi, Korea) was 97% pure quercetin which was extracted from the flower of QS.

The birds were housed in 3-floor battery cages (1.55×0.75 ×0.55 m/cage), in an environmentally controlled room (temperature was started at 32°C and reduced by 2°C every week up to 24°C, and 65% relative humidity). The lighting program was 24 h a day for the first week (days 0 to 7) and then reduced to 16 h of light and 8 h of dark during 7 to 35 days. Each cage was equipped with 2 feeders (one feeder on each side) and 2 nipple drinkers to provide feed and water *ad libitum* to birds. Diets were formulated to meet the nutrient requirements recommended by the NRC (Table 1) [20] and provided in mash form.

### Sampling and measurements

Body weight and feed intake (FI) in each replicate cage was recorded on the 0th, 7th, 21st, and 35th day to measure the body weight gain (BWG), FI, and feed conversion ratio (FCR). For deaths during the middle of a weighing period, the weight of dead animal was recorded, and the gain of the dead bird was counted towards pen gain in figuring feed conversion. The numbers of dead birds were examined as well.

On the 28th day, 2 g/kg chromium oxide was added to the diet as an inert marker to measure the apparent total tract digestibility (ATTD) of dry matter (DM) and nitrogen (N), and the apparent retention of energy. The representative feed samples were taken after proper mixing and stored in the −20°C freezer. During days 33 to 35, excreta samples were collected from each replicate cage in duplicates and stored in a freezer at −20°C until analysis. On day 35, feed and excreta samples were thawed and dried at 60°C for 72 h, then finely ground to pass through a 1-mm sieve and collected. Following the procedure established by the Association of Official Analytical Chemists (AOAC International) [21], diet samples were analyzed for DM (method 930.15), N (method 968.06), crude fiber (method 991.43), calcium (method 984.01), phosphorus (method 965.17), crude fat (method 954.02), and crude ash (method 942.05). Excreta powder samples were also analyzed for DM (method 930.15) and crude protein (method 968.06) following the procedures established by AOAC International [21]. The lysine and methionine content of the diets were measured using an AA analyzer (Beckman 6300; Beckman Coulter, Inc., Fillerton, CA, USA). The combustion heat was measured by a bomb calorimeter (Parr 6100; Parr Instrument Co., Moline, IL, USA) to determine the gross energy content of the feed and excreta powder samples. Chromium concentration was determined by atomic absorption spectrophotometry (UV-1201, Shimadzu, Kyoto, Japan). The equation for calculating digestibility was as follows:


$$Digestibility\;(\%)=\left(1-\frac{Nf\times Cd}{Nd\times Cf}\right)\times 100$$

where Nf = nutrient concentration in excreta (% DM), Nd = nutrient concentration in diet (% DM), Cf = chromium concentration in excreta (% DM), and Cd = chromium concentration in diet (% DM).

At the end of the experimental period, 68 birds (4 birds per replicate cage) were randomly selected from each treatment and blood samples were collected from the wing vein using a sterile syringe and stored at 4°C. Samples for serum analysis were then centrifuged at 3,000×*g* for 15 min at 4°C. Total cholesterol, triglyceride, low-density lipoprotein cholesterol (LDL-C) and high-density lipoprotein cholesterol (HDL-C) concentrations in serum were determined enzymatically using reagent kits (Wako Pure Chemical Industries Ltd., Tokyo, Japan).

After blood collection, they were slaughtered by cervical dislocation. The breast muscle, liver, abdominal fat, bursa of fabricius, and gizzard were removed and weighed to calculate the relative organ weight. The breast muscle was removed and stored at 2°C for the measurement of meat quality. The organ index was calculated using the following equation:


$$Organ\;index=\frac{Organ\;weight}{Live\;body\;weight}\times 100\%$$

After slaughtering the birds, fresh digesta content samples were collected from the caecum into micro-tubes. One gram of digesta sample was blended with 9 mL sterile peptone water and mixed for 1 min on a vortex stirrer. Counts of viable bacteria in the caecum samples were determined by plating serial 10-fold dilutions (10^−3^ to 10^−6^) onto Lactobacilli MRS agar (Difco Laboratories, Detroit, MI, USA), MacConkey agar (Difco Laboratories, USA), and Salmonella-Shigella (SS) agar (Difco Laboratories, USA) plates to isolate lactic acid bacteria, coliform bacteria, and Salmonella, respectively. The lactobacilli agar plates were then incubated for 24 h at 37°C under anaerobic conditions. The MacConkey and SS agar plates were incubated for 24 h at 37°C under aerobic conditions. After the incubation periods, colonies of the respective bacteria were counted and expressed as the logarithm of colony-forming units per gram (log_10_ CFU/g).

The pH values of each breast meat sample were measured in duplicate using a pH meter (Fisher Scientific, Pittsburgh, PA, USA). Thereafter, a 0.20 g meat sample was pressed at 20.7 MPa for 3 minutes on a 125-mm-diameter piece of filter paper. The areas of the pressed sample and the expressed moisture were delineated and then determined using a digitizing area-line sensor (MT-10S; M.T. Precision Co. Ltd., Tokyo, Japan) to calculate the water holding capacity (WHC). About 5 g of meat sample was suspended in a zipper bag in a 4°C environment and weighed on the 1st day to calculate the drip loss.

### Statistical analysis

All data were subjected to statistical analysis in a randomized complete block design using the General Linear Model procedure (SAS Inst. Inc., Cary, NC, USA). The replicate cage was used as the experimental unit. Orthogonal contrasts were used to examine the linear and quadratic effects in response to increasing the dietary supplementation of QS. Variability in the data was expressed as the standard error of means, p<0.05 is considered statistically significant.

## RESULTS

The BWG during days 0 to 7 (p = 0.021), 7 to 21 (p = 0.010), and 1 to 35 (p = 0.045), and FI during days 0 to 7 (p = 0.037) and 1 to 35 (p = 0.025) increased linearly with the increase in the QS levels in the diet. However, the FCR did not differ among all dietary groups (Table 2).

Supplementing graded levels of QS to the diet of broiler chicks linearly increased apparent DM digestibility (p = 0.008) and apparent energy retention (p = 0.004), whereas it did not affect the apparent nitrogen digestibility (Table 3).

Dietary supplementation of QS had no significant effects on the serum total cholesterol, triglyceride, HDL-C, and LDL-C concentrations (Table 4).

The counts of lactic acid bacteria (p = 0.023) increased linearly as the dose of QS increased in the diet. However, the coliform bacteria and *Salmonella* counts in cecal contents were not differ among all dietary groups (Table 5).

Broiler chicks fed the diet supplemented with QS linearly increased the relative weight of breast muscle (p = 0.014), while liver, abdominal fat, bursa of fabricius, and gizzard were not affected, with the increase of the QS dosage. In addition, supplementing graded levels of QS to the diet of broiler chicks linearly increased pH value (p<0.001) and WHC (p = 0.012) from breast muscle, while linearly decreased drip loss (p = 0.001) from breast muscle as the dose of QS increased (Table 6).

## DISCUSSION

Reports on the effects of dietary supplementation of quercetin on the growth performance of broiler chicks were inconsistent. Goliomytis et al [12] reported that feeding broiler chicks with 0.5 or 1.0 g/kg quercetin dihydrate compound containing diet had no effects on the BWG and FI, but increased FCR. In addition, many studies have reported that the growth performance was not affected in broiler chicks fed with a chemically synthesized quercetin containing diet [13,14,16,22]. Conversely, Abid et al [18] reported that supplementing 0.1, 0.2, or 0.3 g/kg quercetin extracted from the locust tree to the diet of broiler chicks had positive effects on the growth performance. Sohaib et al [19] fed broiler chicks with 0.1, 0.2, or 0.3 g/kg plant-derived quercetin containing diet and found an increase of BWG and a decrease of FCR compared with those fed with the control diet. Zhang and Kim [17] observed the increase of BWG in broiler chicks fed the diet supplemented with 0.25, 0.50, or 1.0 g/kg plant-derived quercetin. In the present study, the improvement of growth performance was also observed in broiler chicks fed with QS containing diet. The different effects of quercetin supplementation on the growth performance of broiler chicks may be due to the different sources of quercetin. According to the report of Rasouli and Jahanian [23], the improvement of growth performance in broiler chicks could be achieved by regulating the intestinal microbiota community.

It is well known that the distribution and quantity of intestinal microbiota communities directly affect the health and growth of broiler chicks [24]. A high level of intestinal beneficial bacteria is beneficial to improve nutrient digestibility [24]. Abolfathi et al [25] reported the strategy for improving the nutrient digestibility by increasing intestinal lactic acid bacteria counts in feeding broiler chicks with flavonoid-enriched herbal extract, thus improving growth performance. In this study, we also observed the improvement of apparent DM digestibility, energy retention, and the counts of cecal lactic acid bacteria by QS supplementation. It has been reported that the quercetin and lactic acid bacteria co-cultured *in vitro* showed that quercetin increased the hydrophobicity of cell surface and improved the auto-aggregation and coaggregation capacity of lactic acid bacteria [26]. The hydrophobicity and aggregation of cell surface are related to the ability of probiotics to adhere to the intestinal mucosa [26,27], which means quercetin has modulatory effects on lactic acid bacteria. Studies on the metabolism of quercetin and flavonoids *in vivo* of broiler chicks, humans, and rats have shown that dietary quercetin is absorbed in a small percentage (below 10%) in the small intestine due to the high hydrophobicity of quercetin and the high hydrophobicity of the intestinal mucus layer. The rest of these compounds reach the colon and exert biochemical activity in an unabsorbed form [6,28–30]. Therefore, the lactic acid bacteria modulation effects of quercetin as mentioned above possibly occurred in the colon. In addition, quercetin has been reported to exert potential prebiotic effects *in vivo* [31], thus promoting the growth of intestinal beneficial bacteria [24]. Therefore, the improvement of the counts of lactic acid bacteria by quercetin supplementation is probably related to its lactic acid bacteria modulatory ability and the prebiotic effects. Similar to our results, Zhang and Kim [17] reported that broiler chicks fed the diet supplemented with 0.25, 0.50, or 1.0 g/kg plant-derived quercetin increased cecal lactic acid bacteria counts, but the coliform bacteria and *Salmonella* counts in cecal contents were not affected. Wang et al [24] noted that supplementing 0.2 g/kg chemically synthesized quercetin to the diet of broiler chicks increased cecal lactic acid bacteria counts. In addition, Zhao et al [32] found that dietary supplementation of quercetin-containing antioxidant substance complexes could reverse the decrease of lactic acid bacteria in the intestine of broiler chicks caused by feeding with a high-fat diet. Therefore, we considered that the improvement of apparent DM digestibility and energy retention were related to the increase of the counts of cecal lactic acid bacteria in broiler chicks fed with QS containing diet, thus benefiting to the improvement of growth performance.

Moreover, Yang et al [33] mentioned that feeding birds with quercetin containing diet increased serum growth hormone (insulin-like growth factor-1) concentrations. Dong et al [34] mentioned that the antioxidant status of broiler chicks improved by quercetin supplementation. Further experiments are needed to explore the effects of dietary supplementation of QS on serum growth hormone concentrations and the antioxidant parameters, so as to reveal the endocrine mechanism of QS supplementation promoting the growth performance of broiler chicks. In brief, the growth promoting effect of QS supplementation in broiler chicks was confirmed in this study.

Too much abdominal fat negatively affected the commercial value of the carcass of broiler chicks [35]. It has been reported that quercetin has properties in fat metabolism regulation [36,37]. The lipid deposition regulation properties of quercetin are achieved by modulating the lipid synthesized in hepatocytes [38]. Quercetin could reduce lipid deposition in broiler chicks by promoting PPARα-regulated lipid decomposition [38], reducing the expression levels of sterol regulatory element-binding protein 1, peroxisome proliferator-activated receptor gamma, and 3-hydroxy-3-methylglutaryl-CoA reductase in the liver [39], and activating P13K/PKB, AMRK, and/or cAMP signal pathway [39–41]. It has been reported that quercetin supplementation reduced the increase of adipose tissue weight induced by feeding with a high-fat diet in rats [32,42,43]. The content of total cholesterol, LDL-C, HDL-C, and triglyceride in serum are key indicators of lipid metabolism [38,44,45]. In this study, the concentrations of serum total cholesterol, LDL-C, HDL-C, and triglyceride were not affected by QS supplementation. Similarly, Yugarani et al [46] found that quercetin supplementation had no significant effects on the serum total cholesterol and triglyceride concentrations in rats fed with a high-fat diet. Egert et al [47] reported that quercetin supplementation had no significant effects on the serum total cholesterol concentrations in humans. Simitzis et al [48] demonstrated that the serum total cholesterol concentrations in laying hens fed with 0.2, 0.4, or 0.8 g/kg quercetin containing diet was not affected. The liver is the site for lipid metabolism, the variation of its weight reflects the level of metabolic activity intensity [49]. In this study, supplementing QS to the diet of broiler chicks had no significant effects on the relative weight of the liver, which probably means that the lipid metabolic activity intensity was not affected by QS supplementation, which was manifested in the stable serum total cholesterol, LDL-C, HDL-C, and triglyceride concentrations. This is probably the reason for insignificant effects on the relative weight of abdominal fat. Similarly, Goliomytis et al [12] reported that supplementing 0.5 or 1.0 g/kg quercetin had no significant effects on the relative weight of liver and abdominal fat in broiler chicks. Therefore, we considered that the QS supplementation may not regulate the relative weight of abdominal fat.

The high relative weight of breast muscle is beneficial to the market value of broiler chicks [50]. In the present study, dietary supplementation of QS increased the relative weight of breast muscle. Similarly, Zhang and Kim [17] also observed the increasing trend of the relative weight of breast muscle in broiler chicks fed with quercetin containing diet. It has been reported that flavonoid supplementation in the diet of broiler chicks could increase breast muscle weight [51]. Chan et al [52] reported that quercetin prevented the muscle wasting induced by trichostatin A. Kamboh and Zhu [53] proved that the flavonoid increased the protein synthesis in muscle. Liu et al [54] mentioned that quercetin promoted the protein synthesis of laying hens *in vivo*. In general, feeding broiler chicks with QS containing diet was beneficial to increase the relative weight of breast muscle, thus may enhance their market value.

Gizzard is one of the main digestive organs as it is involved in grinding and proteolysis [55]. It has been reported that diet supplemented with structural components which stimulate gizzard development improved nutrient availability [55]. In this study, the relative weight of the gizzard was not affected by QS supplementation, but the apparent DM digestibility and energy retention was improved. Parmar et al [15] reported that dietary supplementation of quercetin had no significant effects on the relative weight of gizzard. Therefore, supplementing QS to the diet of broiler chicks may not improve DM digestibility and energy retention by increasing the relative weight of gizzard, which was confirmed by the study of Zhang and Kim [17].

The bursa of fabricius is the important immune organ in broiler chicks [13]. Reduced weight of the immune organs represents immunosuppression, while an increase in the weight of immune organs is a manifestation of the enhancement of immunity [56]. Plenty of studies proved that quercetin supplementation had no significant effects on the bursa of fabricius indexes [16,17,34]. We also observed the same result that relative weight of bursa of fabricius was not significantly reduced by supplementing QS to the diet of broiler chicks.

The breast muscle of broiler chicks is among the most popular meats in the world [57]. WHC and drip loss are the important indexes to evaluate meat quality [58,59]. Moisture loss leads to the loss of soluble flavor substances in meat [60]. It has been reported that the variation of WHC and drip loss were dependent on the pH [61]. Jiang et al [62] reported that flavonoid supplementation resulted in the increase of pH in the breast muscle, which attributed to the improvement of antioxidant status of breast muscle. Several studies have proved that quercetin is deposited in the breast muscle of broiler chicks when they are fed a quercetin containing diet [6,19,63]. This makes it possible for quercetin to have an antioxidant effect on breast muscle. Goliomytis et al [12] reported that due to the accumulation of quercetin metabolites in the body tissues, it had the effect of reducing lipid oxidation rates. Therefore, it is reasonable to consider that the high pH value of breast muscle caused by QS supplementation was related to the improvement of antioxidant status [12,64]. However, further experiments are needed to evaluate the effects of QS supplementation on the antioxidant status of breast muscle. On the other hand, WHC and drip loss are dependent on the content of protein in muscle [65]. The protein synthesis properties of flavonoids in the muscle have been proved by Kamboh and Zhu [53]. Liu et al [54] reported that quercetin could promote the protein synthesis of laying hens *in vivo*. In this study, supplementing QS to the diet of broiler chicks increased the relative weight of breast muscle, which means that the QS supplementation probably increased protein synthesis in the muscle, thus benefiting the improvement of WHC and drip loss. In brief, dietary supplementation of QS is beneficial to increase WHC and decrease drip loss, which probably improves the acceptability of the meat to consumers [66].

## CONCLUSION

Dietary supplementation of QS had positive effects on growth performance, apparent DM digestibility, apparent energy retention, cecal beneficial microbiota counts, breast muscle yield, and breast meat quality. This study confirmed the positive effects of QS on the growth performance of broiler chicks, and indicated that QS has potential for improving the performance and enhancing some aspects of the meat quality in poultry.

## Figures and Tables

**Table 1:** Composition and nutrient levels of the experimental basal diet (%, as-fed basis)

Items	Feeding phases		

	Starter (days 0 to 7)	Grower (days 7 to 21)	Finisher (days 21 to 35)
Ingredients (%)			
Corn	43.63	47.45	53.78
Soybean meal	35.08	31.28	28.18
Corn gluten meal	13.00	13.00	10.00
Wheat bran	3.00	3.00	3.00
Soy oil	1.76	1.74	1.51
Dicalcium phosphate	1.81	1.81	1.81
Limestone	0.94	0.94	0.94
Salt	0.36	0.36	0.36
Methionine (99%)	0.19	0.19	0.19
Lysine	0.03	0.03	0.03
Mineral mix^1)^	0.10	0.10	0.10
Vitamin mix^2)^	0.10	0.10	0.10
Total	100.00	100.00	100.00
Calculated value			
Metabolism energy (MJ/kg)	13.40	13.40	13.40
Available P (%)	0.54	0.53	0.52
Analyzed composition			
Crude protein (%)	23.00	21.50	20.00
Ca (%)	1.10	1.08	1.07
Total P (%)	0.83	0.82	0.79
Lys (%)	1.26	1.15	1.06
Met (%)	0.54	0.52	0.50
Crude fat (%)	4.45	4.51	4.32
Crude fiber (%)	3.55	3.48	3.30
Crude ash (%)	6.76	6.57	6.30

1) Provided per kg of complete diet: 37.5 mg Zn (zinc sulphate); 37.5 mg Mn (manganous oxide); 37.5 mg Fe (iron sulphate); 3.75 mg Cu (copper sulphate); 0.83 mg I (potassium iodide); and 0.23 mg Se (Sodium selenite).

2) Provided per kg of complete diet: 15,000 IU Vit A (acetate), 3,750 IU Vit D_3_, 37.5 IU Vit E (α-tocopherol acetate), 2.55 mg Vit K_3_ (MSB), 3 mg thiamin, 7.5 mg riboflavin, 4.5 mg Vit B_6_ (pyridoxin-HCl), 24 ug Vit B_12_, 51 mg niacin (niacinamide), 1.5 mg folic acid (crystalline), 0.2 mg biotin, and 13.5 mg Ca-Pantothenate.

**Table 2:** Effect of dietary supplementation of quercetin extracted from the flower of *Sophora japonica* (QS) on growth performance of broiler chicks

Items	QS (g/kg)				SEM	p-value	
	
	0.0	0.2	0.4	0.6		Linear	Quadratic
Initial body weight (g)	42.75	42.88	43.03	43.34	0.288	0.151	0.751
Body weight gain (g)							
Days 0 to 7	125.93	129.16	132.25	132.21	1.959	0.021	0.414
Days 7 to 21	650.48	667.19	662.75	673.27	5.010	0.010	0.544
Days 21 to 35	939.63	988.80	960.52	987.32	20.269	0.220	0.587
Days 0 to 35	1,716.03	1,785.15	1,755.52	1,792.80	20.948	0.045	0.456
Feed intake (g)							
Days 0 to 7	148.33	147.04	156.94	153.70	2.609	0.037	0.713
Days 7 to 21	1,004.74	1,010.83	1,015.35	1,033.50	12.582	0.122	0.637
Days 21 to 35	1,710.14	1,768.96	1,744.97	1,761.56	18.865	0.138	0.276
Days 0 to 35	2,863.21	2,926.83	2,917.27	2,948.76	22.736	0.025	0.488
FCR							
Days 0 to 7	1.18	1.14	1.19	1.16	0.023	0.997	0.735
Days 7 to 21	1.55	1.52	1.53	1.54	0.021	0.896	0.453
Days 21 to 35	1.82	1.79	1.82	1.79	0.030	0.514	0.939
Days 0 to 35	1.67	1.64	1.66	1.65	0.013	0.389	0.664
Mortality (%)	0.03	0.03	0.03	0.03	0.016	-	-

SEM, standard error of the mean; FCR, feed conversion ratio.

**Table 3:** Effect of dietary supplementation of quercetin extracted from the flower of *Sophora japonica* (QS) on apparent nutrient digestibility of broiler chicks (%)

Items	QS (g/kg)				SEM	p-value	
	
	0.0	0.2	0.4	0.6		Linear	Quadratic
DM	70.06	70.47	71.38	72.93	0.759	0.008	0.451
Nitrogen	63.70	65.94	65.29	66.47	1.030	0.104	0.611
Energy	71.01	71.47	71.96	73.73	0.634	0.004	0.307

SEM, standard error of the mean; DM, dry matter.

**Table 4:** Effect of dietary supplementation of quercetin extracted from the flower of *Sophora japonica* (QS) on serum cholesterol profiles of broiler chicks

Items	QS (g/kg)				SEM	p-value	
	
	0.0	0.2	0.4	0.6		Linear	Quadratic
Total cholesterol (mg/dL)	117.58	116.25	116.00	120.75	1.908	0.284	0.118
Triglyceride (mg/dL)	28.75	28.25	28.00	26.75	1.650	0.402	0.821
HDL-C (mg/dL)	80.92	80.50	80.50	82.75	1.559	0.434	0.397
LDL-C (mg/dL)	30.92	30.10	29.90	32.65	1.688	0.511	0.297

SEM, standard error of the mean; HDL-C, high-density lipoprotein cholesterol; LDL-C, low-density lipoprotein cholesterol.

**Table 5:** Effect of dietary supplementation of quercetin extracted from the flower of *Sophora japonica* (QS) on cecal microbiota of broiler chicks

Items	QS (g/kg)				SEM	p-value	
	
	0.0	0.2	0.4	0.6		Linear	Quadratic
Lactic acid bacteria (log_10_cfu/g)	7.40	7.58	7.52	7.74	0.091	0.023	0.841
Coliform bacteria (log_10_cfu/g)	6.28	6.18	6.07	6.03	0.133	0.158	0.800
*Salmonella* (log_10_cfu/g)	5.37	5.04	4.96	5.01	0.177	0.148	0.296

SEM, standard error of the mean.

**Table 6:** Effect of dietary supplementation of quercetin extracted from the flower of *Sophora japonica* (QS) on carcass traits of broiler chicks

Items	QS (g/kg)				SEM	p-value	
	
	0.0	0.2	0.4	0.6		Linear	Quadratic
Relative organ weight (%)							
Breast muscle	18.09	19.31	19.15	19.40	0.328	0.014	0.145
Liver	2.60	2.52	2.57	2.54	0.025	0.315	0.291
Abdominal fat	1.14	0.85	1.04	1.00	0.099	0.604	0.190
Bursa of Fabricius	0.21	0.18	0.18	0.18	0.015	0.170	0.414
Gizzard	1.55	1.39	1.54	1.54	0.066	0.694	0.244
Breast muscle quality							
pH value	5.43	5.70	5.65	5.86	0.055	<0.001	0.609
WHC (%)	47.31	52.21	49.16	53.11	1.229	0.012	0.703
Drip loss (%)	2.25	1.99	1.92	1.75	0.095	0.001	0.636

SEM, standard error of the mean; WHC, water-holding capacity.
